# Banking for health: opportunities in cooperation between banking and health applying innovation from other sectors

**DOI:** 10.1136/bmjgh-2017-000598

**Published:** 2018-06-06

**Authors:** Ilona Kickbusch, Rüdiger Krech, Christian Franz, Nadya Wells

**Affiliations:** 1 Global Health Centre, Institut de Hautes Etudes Internationales et du Developpement, Geneve, Switzerland; 2 Organisation mondiale de la Sante, Geneve, Switzerland; 3 CPC Analytics, Berlin, Germany; 4 Institut de sante globale, Universite de Geneve, Geneve, Switzerland

**Keywords:** public health, health systems, health economics

## Abstract

The annual funding need for global health SDG targets is estimated by WHO at US$134 billion per year, rising to US$274-$371bn by 2030. This paper examines the challenge of making sustainable investment structures in global health more attractive for mainstream financial markets. The objective is a framework for targeted future debate with financial sector actors. Four case studies of innovative sustainable investment mechanisms are analysed, elaborating potential transfer of green and impact investment models in order to channel additional private sector funds to health. To increase private sector involvement, profit must accrue to providers of finance. The paper shows how health criteria can be incorporated into structures, which create triple bottom line return opportunities. Health infrastructure projects based on risk sharing models with governments or multilateral agencies could use long-term funding, with better credit ratings and lower cost of capital. Outcomes based investment, similar to green or social impact bonds, with third-party certification of measurable health impact, satisfy the private sector need for return with social interest objectives. Responsible investment could expand by adding a ‘health’ (H) criterion to the Environmental, Social and Governance (ESG) framework, implementing ESG+H for mainstream investment screening. These models are scalable, satisfy the need to dedicate funds to health and incorporate consistent critical success metrics. The conclusion finds that strong legal frameworks and exploration of fiscal incentives will be critical next steps to facilitate scaling up and broadening of interest from private sector financial actors. The impact these investments have on overall population health is a positive externality of sustainable global health investment.

**Key questions**

**What is already known?**

- The annual funding need for global health is estimated at US$134 billion per year potentially rising to US$274bn - $371bn by 2030 under WHO’s scenarios for reaching SDG3.
- The private sector will be critical to filling the financial gap.
- Mainstream financial markets do not have access to scaleable structures which facilitate sustainable investment in global health and allow profit to accrue to the providers of finance.

**What are the new findings?**

- Health criteria can be incorporated into financing structures to create triple bottom line return opportunities.
- Strong legal frameworks and the exploration of fiscal incentives to support creation and scaling up of these structures will be critical to broader interest from financial sector actors.
- Such investments can have a positive impact on overall population health supporting economic growth and sustainability.

**What do the new findings imply?**

- Health infrastructure projects based on risk sharing models with governments or multilateral agencies could use long-term funding, with better credit ratings and lower cost of capital.
- Outcomes based investment, similar to green or social impact bonds, with third-party certification of measurable health impact, satisfy the private sector need for return with social interest objectives.
- Responsible investment could expand by adding a ‘health’ (H) criterion to the Environmental, Social and Governance (ESG) framework, implementing ESG+H for mainstream investment screening.
- These models are scalable, satisfy the need to dedicate funds to health and incorporate consistent critical success metrics.

## Channelling financing activities

Under WHO scenarios for making progress towards achieving health targets under SDG3, an additional US$274-371 billion in annual spending is needed by 2030.^1^ In contrast to green investments, financial actors have not yet found sufficient structures to invest in sustainable health on a broad scale. What is needed is a commitment to make investments in health more attractive to mainstream global financial markets in order to channel private funds to profitable health projects.

This paper describes examples of infrastructure financing, green investment and social impact investment where new frameworks were introduced to deliver ‘a double bottom line’ of profits and social benefits, without necessarily reducing returns. These case studies from other sectors are then applied to health to develop opportunities for the creation of sustainable global health investment structures.

## Innovative infrastructure financing

Infrastructure investment is an interesting case study for health. Estimates of the global investment gap for transport, telecommunications and power supply amount to US$1–US$1.5 trillion per year.^2^ In Europe alone, €200 billion annually is needed to fill this gap^3^ and government budgets remain constrained after the financial crisis in the late 2000s.

At the same time, global funds in sovereign wealth funds, insurance companies, mutual funds and pension funds have accumulated large assets. Lobbying association The City UK estimated that total assets under the management of those investors reached US$101.5 trillion (as a comparison: the total market capitalisation of US listed companies amounted to US$25.1 trillion in 2015.^4^ Hypothetically, if a small fraction of these assets went into infrastructure, the global investment gap could be closed, and these investors seek long-term investments—a characteristic that matches the requirements of infrastructure projects.

In past decades, governments tried to use public–private partnerships (PPPs) to bring together public needs and private investments. However, as shown in online supplementary figure 1 in the annex, private participation in infrastructure projects has been negatively impacted since the economic downturn of 2008. Whereas projects a decade ago may have been larger, today we see higher volumes of smaller value projects in the health sector for example, in energy efficiency. Some of the key obstacles of those PPPs have been the complicated contractual arrangements, long payback periods, exposure to political risk^i^ and the need for solid credit ratings notwithstanding questions raised about the role of rating agencies after the 2008 crisis.

10.1136/bmjgh-2017-000598.supp1 Supplementary file 1

**Figure 1:**
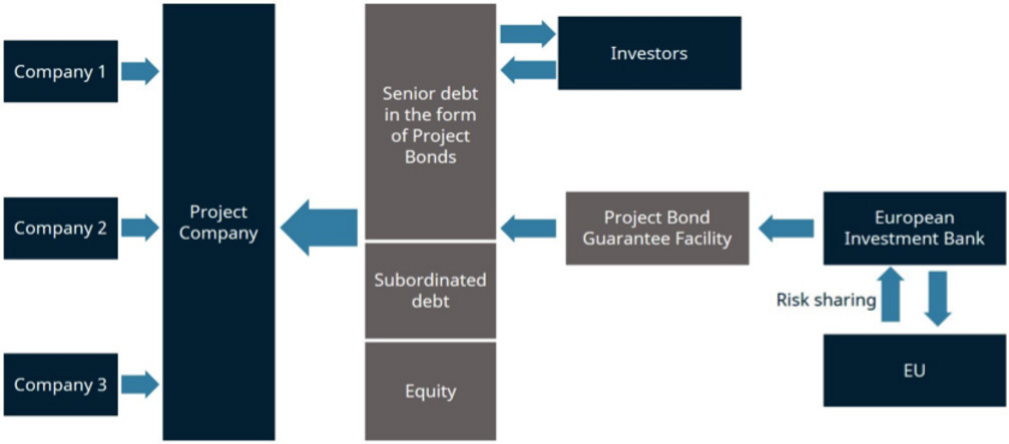
Structure of the Europe 2020 Project Bond Initiative. Note: Illustration adapted from IOSCO (2014)^6^. EU, European Union.

Thus, it is not surprising that over the past 5 years there have been significant efforts to innovate in infrastructure financing. One such example is the Project Bond Initiative (PBI) of the European Union, which started in 2012 and aims to expand capital market financing of large European infrastructure projects as illustrated in figure 1 given below:

At first, one or more companies set up a firm that plans, constructs, operates and finances an infrastructure project. The European Investment Bank will provide subordinated debt instruments—either a loan or a letter of credit—to enhance the credit quality of senior debt issued by the project finance issuers. The goal of this step is to make issued debt securities of the project company more attractive to institutional investors, such as pension funds, that get a stable income with a good risk–return profile. Thus, the investment’s overall cost of capital for infrastructure investment can be lowered.^5^ In the medium term, there are further opportunities: if the PBI works, it can enable the establishment of a platform or a new asset class with participation from private investors.^6^

What makes this approach interesting for health is the idea that governments and banks can help to create a bond market for health infrastructure projects. Hospitals, elderly care homes, healthcare centres require large investments that will pay off only in the long term. A key element is the risk sharing between the sovereign and the bank that issues the project bond guarantee facility. When risk and return are in balance, financial resources follow. The credit enhancement might not be available to governments, which are themselves less favourably rated, although as the example below shows, World Bank/multilateral agency involvement can mitigate this issue.

### Translating into the health arena

A promising project is the Global Financing Facility (GFF) supporting improvement of reproductive, maternal, newborn, child and adolescent health. Launched in July 2015, the GFF Trust Fund has received pledges of US$800 million from the governments of Norway and Canada. In order to increase private sector involvement, GFF’s business plan also includes the possibility to use the World Bank’s AAA credit rating to issue a bond that would allow large-scale mainstream investment.^7^ Private sector investors could get exposure to project finance within a low/middle-income country through a fixed-income asset.

## Green bonds

Introduced with the goal of channelling global funds from a wide range of investors into projects related to climate change, green bonds have made it from niche product to the attention of the mainstream. The key difference from a traditional bond is that the project for which the money is raised is fixed to purposes that protect the environment. Importantly, a third-party certification agent checks whether the investment actually fulfils this claim.

*The problem is not a lack of interest but ‘a lack of product,’ said Matt Arnold, managing director of environmental affairs at JPMorgan Chase*.^8^

**Figure 2:**
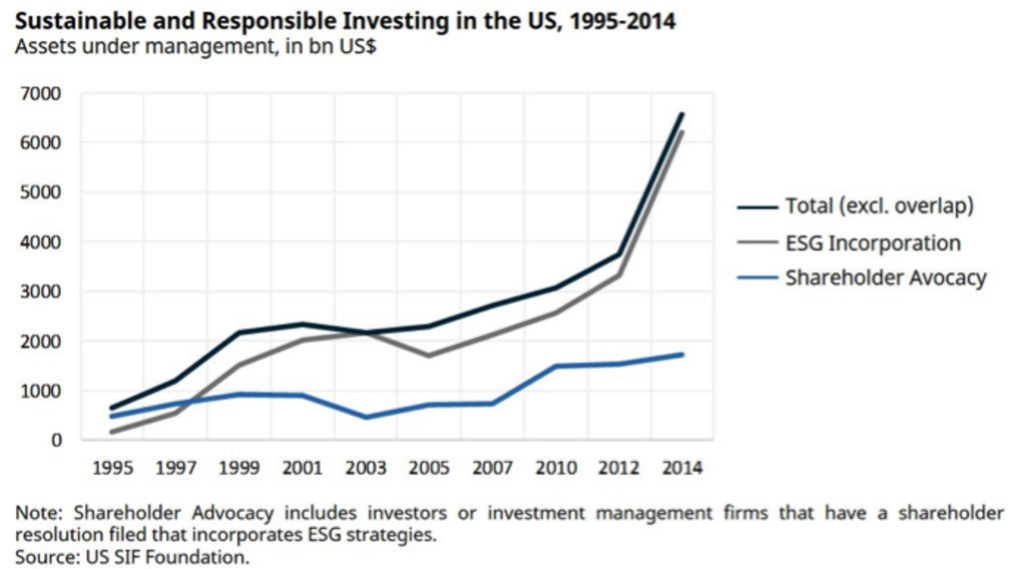
Sustainable and responsible investing in the USA during 1995–2014.

In November 2008, the World Bank, in collaboration with Swedish banking group SEB, started issuing green bonds to finance its environmental projects. The International Finance Corporation issued US$1 billion of green bonds in February of 2013 to finance renewable energy, sustainable transportation and other environmentally friendly projects.^9^ Later the same year, French energy company Électricité De France (EDF) raised €1.4 billion to finance 13 renewable energy projects.^10^ In the following year, financial institutions such as the Bank of America issued green bonds and S&P launched the Dow Jones Green Bond Index. In 2014, new issuance was more than three times the previous year.^11^ Issuance from 2007 to 2015 raised US$100 billion (figure 2).^12^

In its early stages, the market was dominated by international organisations such as the World Bank and other public financial institutions. Currently, big corporates (EDF, UNILEVER) and financial institutions share the market. Overcoming governance issues has been crucial. The Green Bond Principles (GBP),^13^ established in 2014, promote integrity of the market by setting guidelines on transparency, which is further guaranteed through regular project reporting. Eligibility of projects is also guaranteed by criteria predefined and verified by environmental specialists. An example is the US$1 billion refinancing of an investment into offshore windmills in the German North Sea by the US-based private equity investor Blackstone through issuing several different green bonds.^14^

The role of the private sector from a Goldman Sachs report^15^:

***Role of private capital***: Protecting nature is capital-intensive work, and the scale of environmental problems is too large to be solved through philanthropic capital alone. As such, many organizations are looking at innovative models for funding large-scale green infrastructure solutions in partnership with the private sector. TNC, for example, has launched water funds across Latin America to pay for watershed protection and reforestation. Water users contribute to the funds in exchange for fresh, clean water. The funds, in turn, pay for forest conservation efforts along rivers, streams and lakes to help ensure a safe supply of drinking water. TNC’s Quito Water Fund preserves the watersheds that supply the city’s tow million residents with 80 percent of their freshwater. From TNC’s initial $20 000 investment in 2000, monthly contribution from Quito’s water and electric companies now produce nearly $1 million annually in disbursement for conservation projects for the city’s watersheds. TNC is now applying this model to other water fund projects.

The geographical spread has increased. The first Brazilian, and first food sector, green bonds were issued by the biggest Brazilian food company.^16^ The Inter-American Development Bank and the Clean Technology Fund are joining forces to raise US$125 million worth of green bonds for loans on energy efficiency projects in Mexico. The People’s Bank of China issued a Directive on Green Financial Bonds that sets standards on the usage of green bonds.

### Translating into the health arena

The case of green bonds highlights ‘double bottom line’ investment instruments. In health, innovative solutions already exist on a large scale for vaccination programmes: The International Finance Facility for Immunisation (IFFIm) was created in 2006 to provide funds to Gavi, the Vaccine Alliance, as a multilateral institution PPP. ‘The IFFIm operates by issuing bonds, backed by sovereign government commitments…. This not only front-loads Gavi’ s funds, but also enhances its ability to provide multi-year grants to recipient governments.’^17^ Since its inception until 2014, IFFIm has raised US$5 billion for Gavi via selling bonds in international capital markets.^18^ In future we could imagine such structures being used for research and development in pharmaceutical public goods or for investment in medicines for low/middle-income countries.

## Social impact bonds as a variation of impact investment

**Figure 3:**
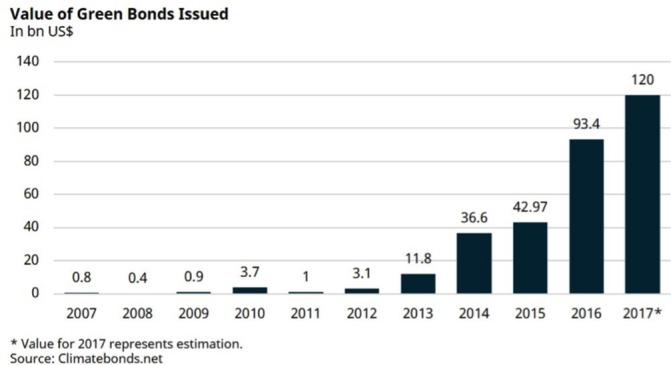
Green Bond Issuance during 2007–2017.

Similar to green bonds, social impact bonds aim to achieve both financial and social returns, but volumes to date have been relatively low. While green bonds help organisations or governments to raise money for a dedicated project, social impact bonds are a tool to finance social services and pay for performance (figure 3).

The fundamental idea behind all outcomes-based investment vehicles is similar: an investor’s returns are dependent on the achievement of a measurable social goal or the improvement of a social indicator. Private investors fund the delivery of a public service upfront and commission the service delivery to a third-party entity. If the service delivery is done with less investment than under the previous government management, the savings are shared between the government and the private investor.

The complexity of these structures mean that they have been heavily scrutinised in order to combine a private sector need for return with social or public interest objectives. In addition, the risks around the evaluation stage related to costs or governance also need to be mitigated upfront. Feasibility depends on four criteria^19^: first, the outcomes that the investor needs to achieve are meaningful and measurable. Second, the achievement needs to be possible in a reasonable time horizon. Third, success must be linked to the interventions funded by the impact bond. Fourth, appropriate legal and political conditions need to be in place.

The Social Impact Investment Taskforce was established in 2014 under UK G8 presidency and delivered a report suggesting that a new investment paradigm has begun.^20^ Similarly, in 2015, the OECD published a summary report covering the major developments in the market.^21^

As the public service that is to be delivered is likely to be uncorrelated with other market developments, social impact bonds could contribute to diversified portfolio performance.

### Translating the learnings into health

In particular, health promotion and prevention programmes are attractive fields. In 2007, the UK Department of Health launched the Social Enterprise Investment Fund, which has invested more than £110 million in the health and social care sector including Social impact bond projects such as the Multidimensional Treatment Foster Care for Adolescents programme providing behavioural interventions for 95 children aged 11 to 14 years^22^. There is also debate around the feasibility of applying social impact investing to finance development projects. For example, a working group at the Washington, DC-based think tank, Center for Global Development, evaluated the opportunity of social impact bonds in six case studies—among those antiretroviral treatment as prevention of HIV and tuberculosis in Swaziland.^23^

The health section of the Global Business Council suggested another idea combining pay-for-performance from social impact bonds with a scalable platform—the so-called Health Credit Exchange (HCX). This platform wants to bring together donors, governments, private sector partners and programme partners (implementation agencies). Companies can invest in health programmes featured on the HCX by purchasing credits. Credits cost US$1, can be purchased at any time, and companies receive a tax benefit at the time of purchase. With those credits, projects on the platform can be supported. The funds are accumulated in a Donor-Advised Fund that will withhold the money until performance metrics are achieved. Interestingly, this allows the funds to be invested in socially responsible stocks of listed firms. As soon as performance metrics are achieved and verified, funds will be paid to the implementing agency. Projects that apply for funding at the GFF will possibly also attract investors from the HCX.^24^

## Changing the investment framework

Legal and fiscal frameworks can help to support interesting new products with social impact. Integration of measurable indicators for the impact of the investment on the ecological and social environment is needed to align incentives of investors and society. The 2016 Annual Impact Investor survey is indicative of the market showing an additional US$15 billion committed during 2015, of which half was invested in low/middle-income countries.^25^ J.P. Morgan and the Global Impact Investment Network forecasted growth of nearly 16% in the amount invested by the 158 leading impact investors in 2016.

Existing corporate governance codes and Environmental, Social and Governance (ESG) criteria could expand to include health impact criteria, ESG+H. Having health criteria included in annual reporting, listing requirements or prospectuses in debt and equity issuance, for example, could add ‘H’ to the ESG asset class.

### Responsible investments more mainstream …

**Figure 4:**
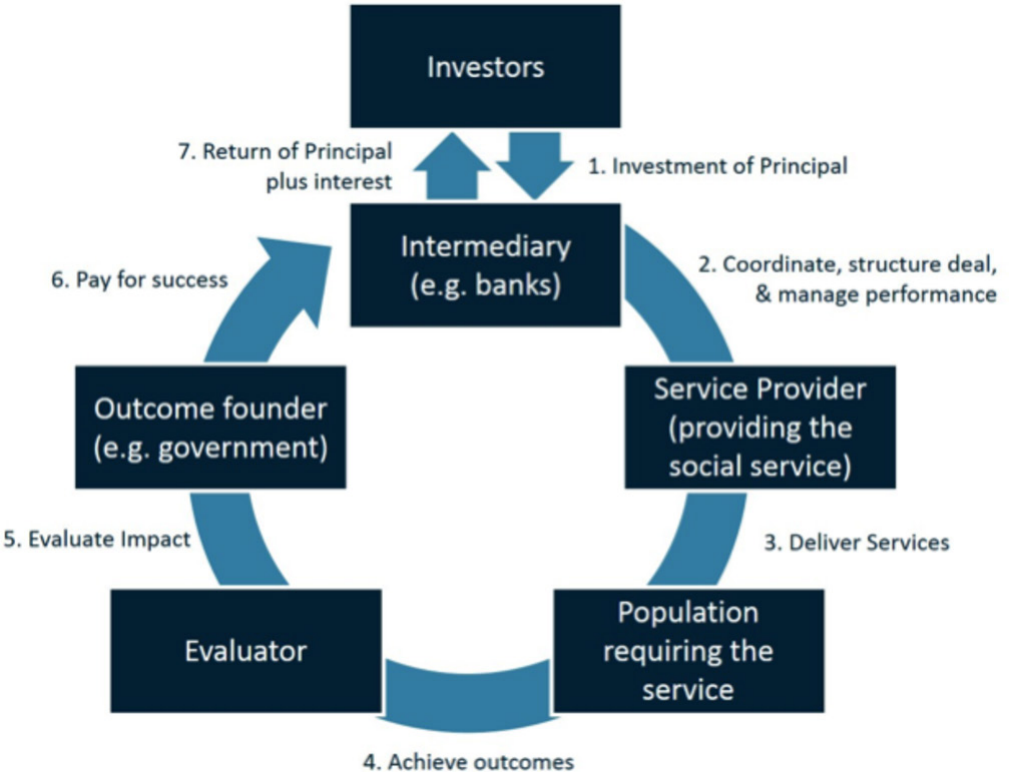
Concept of a possible social impact bond setting (Gustafsson-Wright *et al*).^19^

Figure 4 shows the growing volume of assets under management in the USA guided by ESG criteria, from US$1.2 trillion in 1997 to US$6.6 trillion by 2014,^26^ with an increase of 76% between 2012 and 2014. In the past, those funds were from family offices, high net worth individuals and development finance institutions. Nowadays, banks, pension funds and insurance companies are joining in.^27^

### … also driven by higher customer demand, …

In a survey of 129 US-based money managers, 72% mentioned both client demand and mission as the prime reason for incorporation of ESG criteria.^28^ Interestingly, two-thirds also mentioned risk and return considerations, a sign that incorporation of ESG criteria is becoming more mainstream. This change is also reflected by a subsidiary of Man Group PLC, the world’s largest publicly traded hedge fund: ‘There is a [view] … that hedge funds and responsible investment should not go together in the same sentence…. As the industry has matured and institutionalised, we the asset managers have become increasingly responsive to the requests from the asset owner side—we do owe our ultimate duties to you’.^29^ Indications that the current Chinese government wants to push harder to make companies more compliant with ESG criteria^30^ show that this is not just a developed market phenomenon.

### … leading to a change in investment screening procedures

Adopting an additional ‘health’ criterion to the ESG framework, ESG+H, could help banks and asset managers create mainstream socially responsible products and access new customers.

A key enabler for scalability of sustainable investments is the availability of up-to-date information on ESG indicators by company to allow for appropriate monitoring. A new fund from Blackrock—the largest asset management firm in the world—is an important indicator. Every day, the fund will assess more than 8000 companies and score them based on their impact on health, the environment and corporate citizenship. Additionally, industries that produce harmful services or products are excluded from the start.^31^

Mainstream equity or bond portfolios with a screening overlay allow asset managers to offer daily liquidity, providing potential for larger investment vehicles to be created for a wider range of investors. This is key to bringing these opportunities to a broader audience.

## Towards new instruments for health financing

### Opportunities and challenges of new health financing instruments

This section seeks to summarise a combined framework that can guide future discussion (table 1).

**Table 1:** 

Approach	Opportunities
Innovative infrastructure financing	Raise new funds from private sector investors for large-scale greenfield/brownfield investments by sharing risks with intermediary (eg, reinsurers) which is backed by government funds. creating fixed-income bonds with a good credit rating that are interesting for long-term investors (eg, Sovereign Wealth Funds, insurances, pension funds). Achieve lower cost of capital.
Green bonds	Raise new capital for projects with a specific purpose (eg, climate mitigation) from investors who want to invest in a climate-friendly initiative, without being exposed to risks associated with individual projects. Creates a long-term fixed-income security that can be traded—given a liquid market. First product standardisation efforts have already been made.
Social impact bonds	Raise private funds for (co)financing the delivery of social services. Create investments that do not correlate with mainstream investments which allows diversification of risk.
Business practices	Overcome political constraints to pay for prevention measures (eg, health prevention). Expand risk and return assessment of investments to non-financial dimensions. Enable the creation of new financial products attracting investors who are interested in climate-friendly/health-friendly investments. Expand ESG criteria to ESG+H including health thereby broadening the asset class to a wider group of mainstream investors.
**Approach**	**Challenges**
Innovative infrastructure financing	Good credit rating of the sovereign that backs the bond might limit the availability of the instrument to more creditworthy countries. Each project depends on a variety of different risk factors (competition, security package, counterparty risk, technical risks, availability of labour and materials and event risks) which might make standardisation difficult. Regulatory framework might have to be adapted.
Green bonds	Bonds need to ensure to have investment grade, that is, a good rating that allows institutional investors to integrate the bond into their portfolio. Scalability of such bonds still needs to be proven: sufficient availability of suitable projects and liquidity as the market is still relatively small).
Social impact bonds	Low liquidity: At the moment, there is no secondary market for social impact bonds.
Business practices	Measurability of social outcomes limits the range of suitable projects. Pay for Performance scheme introduces additional risk. Distrust in the market to incorporate non-financial indicators into risk assessment. Need to formulate key health criteria for incorporation in ESG+H framework. Need to build framework for health criteria inclusion in corporate reporting, stock exchange criteria and responsibility frameworks.

ESG, Environmental, Social and Governance; H, health.

### Features of new instruments in health financing

First, often banks and other financial institutions are confronted with barriers to enter the health sector such as specific rating or liquidity requirements. Risk sharing mechanisms such as those used in the Project Bond Initiative in the European Union, can help to create an attractive risk profile for health infrastructure investments. By creating project bonds that have solid credit ratings, the pool of institutional investors that are interested can be increased significantly. A complementary step involves the ‘repackaging’ of investments into products, which can then be distributed to a broader group of investors enabling better risk diversification, both for the banks creating the product and for investors who could access an interesting new asset class.

Scalability depends on the availability of suitable projects and the existence of a liquid secondary market. To create the latter, a certain level of standardisation of the bonds needs to be achieved. With regards to green bonds, this process has already started with the creation of the GBP in 2014.

Second, to make health investments more mainstream, investment products should be designed in a way that clearly dedicates the funds to health. Project monitoring, general practice with green bonds and social impact bonds are crucial. The availability of a credible third-party actor that evaluates the impact is important. In the case of social impact bonds this is particularly crucial as the return is also dependent on the evaluation of the performance of the service delivered.

**Figure 5:**
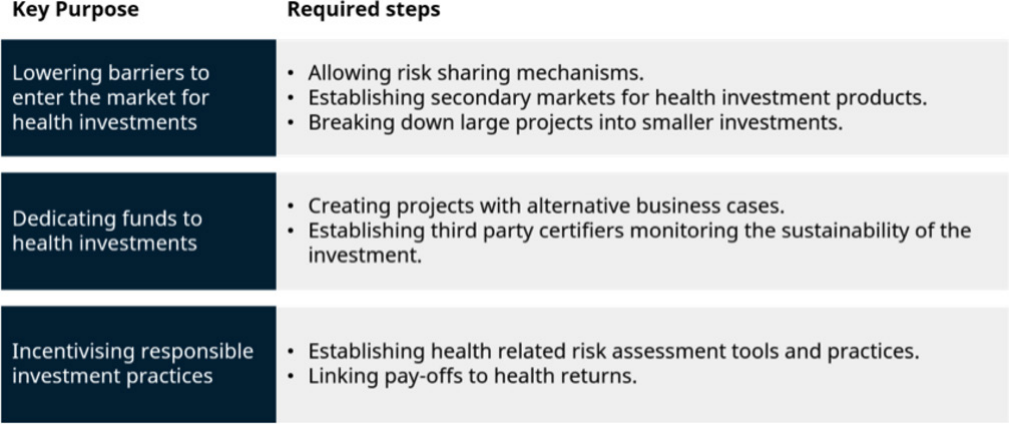
Features of new instruments in health financing.

Third, if banks and other investors recognise health effects of investments in their decision-making process, they will consider these new products in their day-to-day practice. The successful adaptation of the principles of green bonds to ‘health bonds’ and the consistent incorporation of ESG+H criteria into investment strategies are dependent on the willingness of investors to accept health as a crucial success metric (figure 5).

The key challenge for incorporating health criteria is that products need to be created which link the potential financial benefits to those financing, or facilitating the financing, of the project. It is clear that benefits accrue to society as a whole or to reducing government health spending, depending on the structure of the health financing system in the country of investment. However, to increase private sector involvement we need to be innovative in creating opportunities for double bottom line returns from these investments and allowing profit to accrue to the providers of finance.

On the debt side, government or multilateral agency incentives could be envisaged in the form of guarantees to encourage private sector participation. On the investment fund side, fiscal incentives could be envisaged as we see in some countries, which offer lower tax rates for investors in innovation through venture capital or other collective investment instruments.

Climate change mitigation often goes hand in hand with energy saving measures, that is, direct cost saving potential, or was encouraged by the creation of the carbon trading scheme. Thus, investing in a green project brings direct economic benefits to the company that seeks investment. Such arguments might be harder to make for health more broadly where the health status of individuals depends significantly on social and environmental factors. It is also difficult to differentiate growth in the health industry—for example, increased use of certain medications—from its impact on population health.

However, this very impact on overall population health is an added benefit, a positive externality, of sustainable global health investment. This can be most easily seen today in focused areas such as vaccine development. Further research should focus on additional concrete investment opportunities that could be created from putting into practice the principles outlined in this paper.

## Conclusion

This paper aims to create the framework for targeted future debate with financial sector actors on sustainable global health investment structures. New approaches exist, supported by national governments and international institutions: a financial transaction tax to finance health (Brazil), product development partnerships, a levy on airline tickets (UNITAID) and the establishment of the Global Fund for AIDS, Tuberculosis and Malaria. Projects such as the GFF in support of ‘Every Woman, Every Child’, the HCX platform to scale project finance and vaccine bonds through the IFFIm or the new World Bank supported financing mechanism for health emergencies, based on a reinsurance model, provide cases for how to make health financing smart, scalable and sustainable.^32^

Subsequent studies need to pick up the findings from this report to elaborate on a possible transfer of green investment to health investment products and to explore routes such as fiscal incentives for long-term health investment products and the broad introduction of ESG+H criteria. Strong legal frameworks around the deepening of health sector investment will be critical to allowing scaling up and broadening of interest from the financial sector. The close cooperation between bankers, insurers, asset managers and health policy-makers will be crucial for the development of those solutions.

10.1136/bmjgh-2017-000598.supp2 Supplementary file 2

## Footnotes

i Of course, there are many other challenges such as the misalignment of incentives and information asymmetry, exchange rate risks or insufficient expertise of governments
