# Supplementary figures and images for: Banking for health: opportunities in cooperation between banking and health applying innovation from other sectors
Source: BMJ Glob Health. 2018 Jun 6;3(Suppl 1):e000598. doi: 10.1136/bmjgh-2017-000598 (PMC6001912; doi:10.1136/bmjgh-2017-000598)

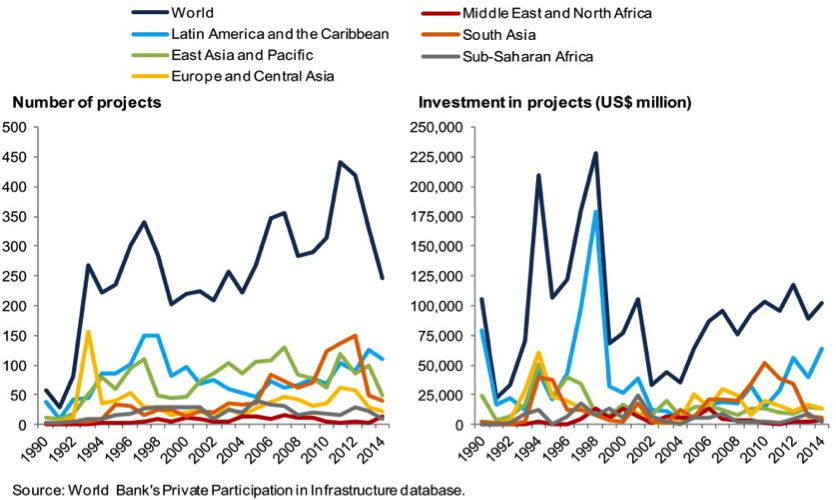

Supplement: Supplementary file 1 [file bmjgh-2017-000598supp001.jpg]
