# Supplementary material for: Banking for health: opportunities in cooperation between banking and health applying innovation from other sectors
Source: BMJ Glob Health. 2018 Jun 6;3(Suppl 1):e000598. doi: 10.1136/bmjgh-2017-000598 (PMC6001912; doi:10.1136/bmjgh-2017-000598)
Supplement: Supplementary file 2 [file bmjgh-2017-000598supp002.docx]

Review of “Banking for Health – Scope for New Partnerships”

Hannah SchiffHannah Schiff

Manager, Research | Global Impact Investing Network

30 Broad Street, 38th floor | New York, NY 10004

hschiff@thegiin.org| Office: +1 (646) 837-7152 | Mobile: +1 (845) 987-6783

**General comments:**

- Translating approaches that have worked elsewhere into the health arena is extremely useful and practical. However, in some cases, the explanation of how it translates to the health sector could be strengthened to include the opportunities that would be enabled (e.g., what health projects or solutions would be financed) and the barriers that would need to be overcome.
- The discussion of risk and return at various points in the paper needs to better acknowledge that these are not objective equations, but rather that different stakeholders have varying preferences and considerations. Risk tolerance and return sought vary from investor to investor. In impact investing, there is a range of return expectations from risk-adjusted market-rate returns to concessionary returns or capital preservation. (E.g., page 3 “when risk and return are in balance…” should better reflect that it’s when the risk and return requirements of an investor are satisfied.)
- The idea of adding “H” to ESG is an interesting one. However, would it make additional funding available for health solutions, or rather would it be more effective at avoiding negative health impacts? I think this is an important distinction to consider.
- The opportunities and challenges table offers a helpful summary, and I think it could be moved up earlier in the paper.
- I think the paper conflates impact investing and ESG screening / management. Impact investing is defined as making investments with the intention to generate a specific positive social or environmental impact alongside financial returns. On the other hand, some investors take into account ESG factors to help them choose companies that adhere to responsible internal business practices – such as fair labor practices, limiting pollution, etc. The approach focused on business practices seems to confuse these.

**Specific comments:**

- On page 7, it says there is a debate around applying social impact investing to finance development projects – but it does not say what the debate is.
- Page 8, the section on changing the investment framework within banks contains some inaccuracies and inconsistencies:
  - If you are going to use figures from our Annual Impact Investor Survey, you should use this year’s report, released in May 2016: <https://thegiin.org/knowledge/publication/annualsurvey2016>
  - This is **not** an estimate of the size of the impact investing market. It captures only a sample of impact investors.
  - Only a small proportion of the respondents are banks, so I am not sure the title of this section is appropriate.
  - The criteria mentioned to participate is incorrect. It’s not only investments above $10 million. It’s just investors that have committed at least $10 million to impact investments – but that might have been through many smaller investments or through larger ones.

**References to be added:**

- The figures in the first paragraph under “Channeling bank activities” need a citation.
- For applying the infrastructure financing model to health, have you looked at the Global Health Investment Fund as an example?
- For social impact bonds: <http://socialfinance.org/social-impact-bonds-the-early-years/>. Can use this to give some overall figures on what’s been done; lessons learned.
- Page 8, you can reference the 2016 Annual Impact Investor Survey (<https://thegiin.org/knowledge/publication/annualsurvey2016>), which includes data on motivations for conventional investors to allocate capital to impact investments on page 49, Table 24. The top motivations are commitment as a responsible investor, efficient way of meeting impact goals, responding to client demand, and an opportunity to gain exposure to growing sectors and geographies.
- You could also reference the healthcare allocations figures from our 2016 Annual Survey. Of the USD 77.4 billion sample AUM in impact investing in 2015, 4% was allocated to healthcare, or just over USD 3 billion. While it represents a small proportion of capital, healthcare is the second-ranked sector in terms of number of respondents investing there; 78 of the 158 investors surveyed have some allocation to healthcare (about half). Healthcare is also a top sector of interest for increasing allocations as a proportion of total portfolios in the coming year, with 41 respondents planning to do so. See pages 17-18.
